# Prophylactic anticoagulation in nephrotic syndrome prevents thromboembolic complications

**DOI:** 10.1186/s12882-019-1336-8

**Published:** 2019-04-25

**Authors:** Sarah Kelddal, Karen Marie Nykjær, Jon Waarst Gregersen, Henrik Birn

**Affiliations:** 1 Department of Renal Medicine, Aarhus University Hospital, Palle Juul-Jensens Boulevard 35, 8200 Aarhus N, Denmark; 2 Department of Internal Medicine, Viborg Regional Hospital, Heibergs Alle 4, 8800 Viborg, Denmark; 3 Accidents and Emergency Department, Herning Regional Hospital, Gl Landevej 61, 7400 Herning, Denmark; 4 Department of Nephrology, Aalborg University Hospital, Hobrovej 18-22, 9000 Aalborg, Denmark; 5 Department of Biomedicine, Aarhus University Health, Vennelyst Blvd. 4, 8000 Aarhus, Denmark

**Keywords:** Nephrotic syndrome, Glomerulonephritis, Anticoagulation, Thromboembolic event, Albuminuria

## Abstract

**Background:**

An increased incidence of thromboembolic events (TE) are reported in nephrotic syndrome (NS) leading to recommendations for prophylactic anticoagulation (PAC). However, as no randomized clinical trial has established the efficacy or risks associated with PAC, guidelines are empiric or substantiated only by estimates of risks and benefits.

This study evaluates the risk of TE and hemorrhagic complications in patients with NS treated with PAC and compares to patients not receiving PAC.

**Methods:**

We included patients diagnosed with NS from two Danish nephrology departments with different practices for the use of PAC. Patients were included if presenting with NS from September 2006 to January 2012, a P-albumin < 30 g/L, and renal biopsy confirming non-diabetic, glomerular disease. Patients aged < 16 years, on renal replacement therapy, or administered anticoagulants at the onset of NS were excluded. Bleeding episodes and/or TE were identified from patient records. Bleeding episodes were divided into minor and major bleeding.

**Results:**

Of the 79 patients included, 44 patients received PAC either as low or high dose low-molecular-weight heparin (LMWH) or as warfarin with or without LMWH as bridging, while 35 did not receive PAC. P-albumin was significant lower in the PAC group compared to those not receiving PAC. Significantly more TEs was observed in the non-PAC group compared to the PAC group (4 versus 0 episodes, *P* = 0.035). The TEs observed included one patient with pulmonary embolism (PE), one with PE and deep vein thrombosis, one with PE and renal vein thrombosis, and one with a stroke. Five patients with bleeding episodes were identified among those receiving PAC, of which two were major and three were minor, while two patients in the non-PAC group experienced a minor bleeding episode (*P* = 0.45 between groups). The major bleeding episodes only occurred in patients receiving PAC in combination with low dose aspirin.

**Conclusions:**

In patients with NS the use of PAC was associated with a decreased risk of clinically significant TE, but may also be associated with more bleeding episodes although not statistically significant. Only patients treated with PAC in combination with anti-platelet therapy had major bleeding episodes.

## Background

Nephrotic syndrome (NS) is associated with an elevated risk of thromboembolic events (TE) [1] leading to increased morbidity and mortality [2, 3]. The incidence of TE in NS has been estimated to 3–44% depending on the localization of thrombosis and the extend of the diagnostic screening [4, 5]. The risk of TE is greatest within the first 3 months, although the risk remains elevated for more than 5 years [1]. The pathophysiology underlying the prothrombotic state is not well understood. Several, essential hemostatic proteins, including antithrombin III, and protein S, may be lost in the urine because of the glomerular leakage associated to the primary glomerular defect [2]. Despite the urinary loss of hemostatic proteins levels of some proteins, including fibrinogen, are increased and correlate with the hypoalbuminemia [6]. It is believed that hypoalbuminemia stimulates the hepatic synthesis of hemostatic proteins to make up for the urinary loss [7].

Membranous nephropathy is associated with the greatest risk of TE at any level of serum albumin compared to minimal change and focal segmental glomerulosclerosis [8]. Other risk factors for venous TE include the severity of NS at presentation, mail gender, increased ionized calcium, and a decrease in plasma antithrombin III [2, 9]. A low serum albumin level is also correlated with an increased risk of TE and a previous study showed a 3-fold increased risk of venous thromboembolic events when serum albumin was below 2.5 g/dL [10]. Established risk factors for arterial TE in NS include age, sex, hypertension, smoking, diabetes and low eGFR [11].

While the increased risk of TE in NS is well established, there are no randomized studies to direct the use of anti-coagulative therapy as primary prophylaxis. Only two published trials have analyzed the effects of prophylactic anticoagulation (PAC) on TE in patients with NS. PAC was administered depending on serum albumin levels and the findings suggested that the use of LMWH prevents TE, but no comparable control group were included in either of studies [12, 13]. Given the limited evidence international guidelines often suggest PAC in selected patients with NS based on decision analysis calculations of risk and benefits [14].

To further quantitate the potential benefits of PAC in NS, this study investigated retrospectively the number and type of TE as well as hemorrhagic complications in a cohort of NS patients treated with PAC and compared this to non-treated patients.

## Methods

### Design and inclusion

A retrospective analysis including incident and consecutive patients diagnosed with NS and a renal biopsy confirming glomerular disease from September 2006 to January 2012 at two Danish Renal Departments. NS was defined as urinary protein excretion greater than of 3.5 g/day and/or a urinary albumin excretion greater than 2.2 g/day or 2200 mg/g creatinine in association with a plasma albumin < 30 g/L [15]. The follow up time were at least 3 weeks. Patients aged < 16 years, with diabetic kidney disease, on treatment with anticoagulants at onset of NS, or on renal replacement therapy were excluded. The study was approved by The Danish National Board of Health and the Danish Data Protection Agency. All data were anonymized prior to analyses and according to Danish regulations, individual patient consent was not required for this study.

### Intervention

There were different practices regarding the use of PAC in the two Renal Departments. At the Department of Nephrology, Aarhus University Hospital all patients with NS and plasma albumin < 20 g/L were treated with PAC based on local guidelines recommending LMWH as the initial treatment followed by warfarin at an INR target of 2–3 as oral maintenance therapy if required. The dosing of LMWH and the monitoring of warfarin therapy was at the discretion of the treating physician; however, biochemical monitoring of LMWH by anti–factor Xa levels was not common practice at the time of the study. In contrast, PAC routinely used at the Department of Internal Medicine, Viborg Regional Hospital irrespective of plasma albumin levels.

PAC involved either warfarin alone, warfarin with bridging using low-molecular-weight heparin (LMWH), low dose or high LMWH. Aspirin was not used as PAC, but patients treated with aspirin prior to the diagnosis of NS continued the treatment when initiated on LMWH and/or warfarin. Aspirin was in general prescribed to prevent cardiovascular disease; however, the exact indication in these 11 patients was not recorded.

### Outcome

Baseline characteristics and outcomes were identified from patient records. Baseline characteristics included demographics, plasma albumin, eGFR, urine protein and/or protein, date of renal biopsy and histological diagnosis. P-creatinine, U-creatinine, P-albumin and U-albumin were all measured using automated, standardized clinical assays. All diagnoses of TE and/or bleeding episodes during follow-up were identified from patient records. The diagnosis and workup for a TE was instigated by clinical suspicion as no systematic screening for TE was performed. The TE identified included pulmonary embolism (PE), deep vein thrombosis (DVT), renal vein thrombosis (RVT), and stroke. Bleeding episodes were divided into major bleedings or minor bleedings based on whether blood transfusion was required or not.

### Data analysis

Baseline demographics and outcomes were compared using Fisher’s exact test for categorical measures and Mann Whitney U-test for continuous variables. Data were described using range or medians [interquartile range (IQR)] and a two-sided *p*-value < 0.05 is considered statistically significant.

## Results

A total of 79 patients presenting with NS due to glomerular disease were included in the study. Forty-four patients received PAC and 35 did not. Based on biopsy findings, 35 patients were diagnosed with minimal change disease, 19 were diagnosed with membranous nephropathy, 7 were diagnosed with focal segmental glomerulosclerosis and 18 were diagnosed with other causes such as amyloidosis or non-specific glomerular disease. No significant differences were identified between groups in terms of gender, age and renal function at baseline (Table 1); however, plasma-albumin was significantly lower in the in the PAC group compared to the non-PAC group, (15 g/L, range 10–23 g/L, vs. 20 g/L, range, 11–29 g/L, *p* < 0.001.

**Table 1:** Baseline data

Demographics	Anticoagulation	Non-anticoagulation	*p*-value
Number	44	35	
Male, n (%)	26 (59)	13 (37)	0.053
Median age (year, range)	43 (17–78)	52 (22–84)	
Median serum creatinine (μmol/L, range)	83 (37–547)	107 (43–208)	
Plasma albumin (g/L, range)	15 (10–23)	20 (11–29)	< 0.001
Duration of treatment (days)	26 (1–350)	–	–
Follow-up time (weeks, IQR)	92 (34–178)	49 (19–98)	0.19

Data represent number, range (minimum and maximum value; IQR). Comparison between PAC and non-PAC was made using, Fischer’s test or Mann Whitney U-test

The PAC regimes varied among patients and included patients receiving warfarin with or without bridging as well as LMWH using variable dosing regimens (Table 2). Only two patients from Viborg Regional Hospital received anticoagulation treatment (plasma albumin levels 16 g/L and 23 g/l, respectively). Eleven patients received anti-platelet therapy and four patients received both anti-platelet therapy and PAC.

**Table 2:** Intervention

Intervention (PAC)	Anticoagulation	Non-anticoagulation	*p*-value
Number	44	35	
PAC treatment: (At initial presentation)			
Low dose LMWH, n (%)^a^	15 (34)	–	–
High dose LMWH, n (%)^b^	7 (16)	–	–
Warfarin with LMWH bridging, n (%)^c^	16 (36)	–	–
Warfarin without bridging, n (%)	6 (13)		
Anti-platelet treatment^d^, n (%)	4 (9)	7 (20)	0.20

Range: Minimum and maximum value; IQR: inter quartile range

^a^Dalteparin ≤5.000 IE/day or Enoxaparin 40 IE/day

^b^Dalteparin ≥7.500 IE/day or Enoxaparin 80 IE

^c^Warfarin in combination with Dalteparin or Enoxaparin in any dose

^d^Aspirin 75 mg daily

None of the 44 patients receiving PAC experienced a clinical TE while four patients (11%) had a diagnosis of TE among the 35 patients not receiving PAC (*P* = 0.035, Fischer’s exact test) (Table 3).

**Table 3:** Thromboembolic events and bleeding episodes

TE and bleeding episodes	Anticoagulation	Non-anticoagulation	*p*-value
Number	44	35	
Time to TE, (days, IQR)		22 (7–220)	
Thromboembolic events (TE), n (%)	0 (0)	4 (12)	0.035
Bleeding episodes, n (%)	5 (11)	2 (6)	0.45
Major, n (%)	2 (5)	0 (0)	0.50
Minor, n (%)	3 (7)	2 (6)	1.00

Major bleeding was only observed in patients on concomitant PAC and anti-platelet treatment

**Fig. 1:**
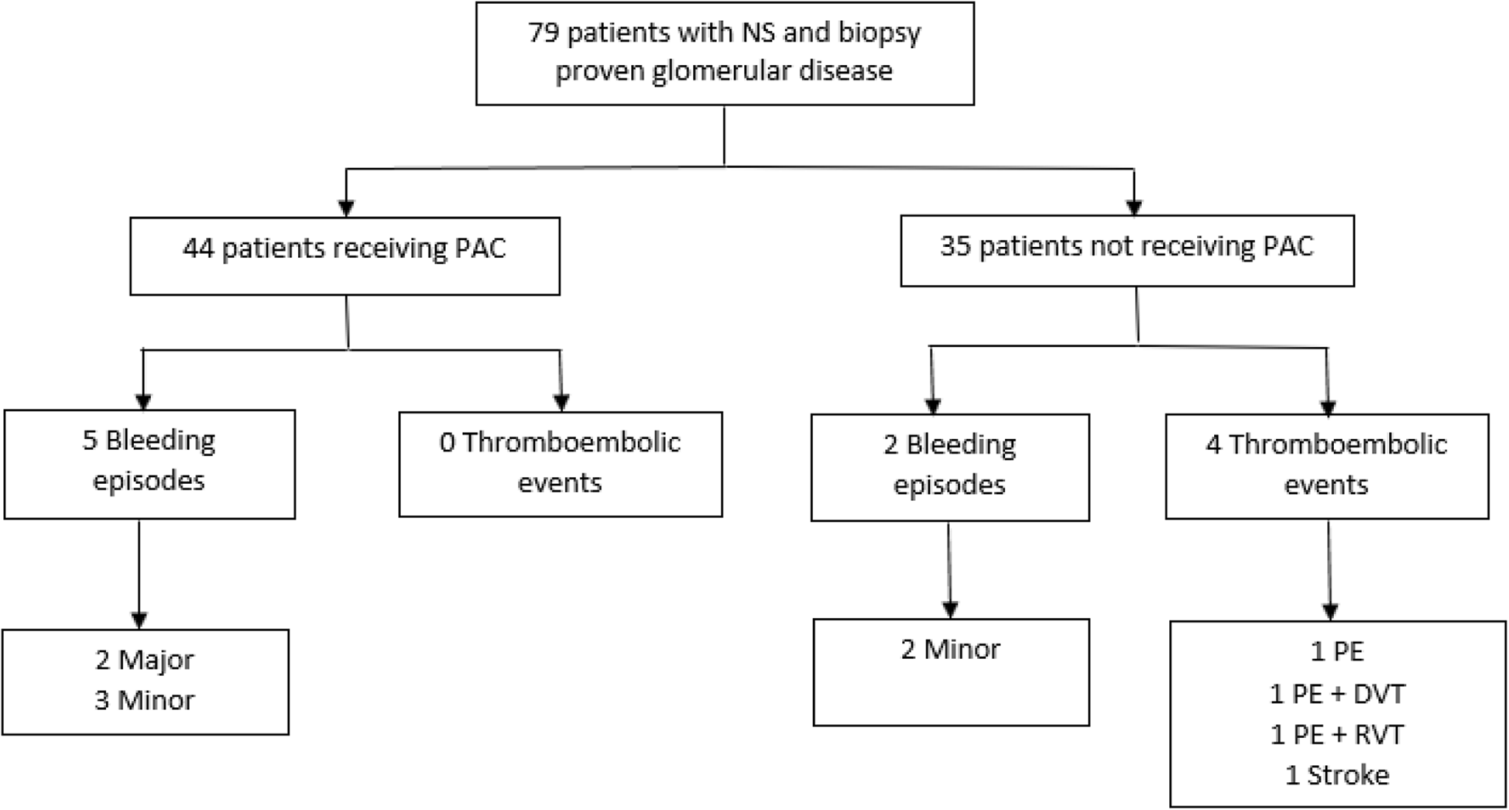
Study overview

The majority of thromboembolic episodes occurred within the first month after the diagnosis of nephrotic syndrome, and included one patient with PE, one patient with PE and DVT, one patient with PE and RVT, and one patient with a stroke (Fig. 1). The glomerular pathology observed in the four patients experiencing a TE was membranous glomerulopathy (*n* = 2), glomerulosclerosis (*n* = 1), and vasculitis (*n* = 1). Five bleeding episodes (11%) were recorded among patients receiving PAC, including two major episodes of gastrointestinal bleeding, of which one was fatal, and three minor. Two minor bleeding episodes were recorded among patients not receiving PAC. This difference was not statistically significant (*P* = 0.45, Fischer’s exact test). Notably, the two major bleeding episodes were observed in patients on warfarin in combination with low dose aspirin. Two of three patients on PAC having a minor bleeding episode were on warfarin and the INR was above target at the time of bleeding (4.5 and 6.7, respectively) while one patient was receiving low dose LMWH.

## Discussion

Based on a retrospective analysis this study shows that the use of PAC to all patients with NS and a P-albumin < 2.0 g/dL significantly reduced the number of clinically significant TE. It was also associated with a non-significant increase in the number of bleeding episodes of which one was fatal.

We are able to compare the PAC-treated NS patients to a group of untreated patients as there were different practices regarding the use of PAC in the two neighboring, renal departments. The two groups were comparable regarding gender, age or renal function; however, plasma albumin was significantly lower in the PAC-group. Different studies have shown that hypoalbuminemia is a major risk factor and strongly associated with venous TE, [9, 16] and thus is possible that the non-treated group was biased against a lower risk of TE suggesting that benefit of PAC in preventing TE may be greater than observed. The incidence of TE in our study, including both arterial and venous thromboembolic events, was 12%. Like previous studies [1] we also found most TE to occur within the first months after the diagnosis of NS.

A total of seven patients experienced a bleeding episode of which five were receiving PAC. Two of these patients had major gastrointestinal bleedings, which required blood transfusion. Both these patients received warfarin in combination with low dose aspirin but unfortunately INR was not recorded at the onset of the bleeding episodes. One of these patients developed circulatory failure and eventually died. Three patients in the PAC group experienced a minor bleeding episode with minor hematomas or a small bleeding in the eyelid. At the time of bleeding, two of the three patients were receiving warfarin with an INR above target range (4.5 and 6.7 respectively). Thus, it is possible that some of these bleeding episodes may be preventable if combinations with aspirin were avoided and a more rigorous control of INR was instituted.

Only two previous studies have examined the association between nephrotic syndrome, thromboembolic events and PAC. In a study including 143 NS patients receiving either Enoxaparin or low dose of aspirin depending on serum-albumin, two cases of PE were identified during the first week of treatment; However, it was not clear if the TE actually happened before the start of PAC. Seven patients were reported to have gastrointestinal bleeding episodes of which five were receiving aspirin and two were on Enoxaparin [12]. A smaller study evaluated 55 patients with NS and a median serum albumin of 17 g/l receiving Enoxaparin 40 IE until urine protein excretion was below 3 g/day [13]. No thromboembolic events were identified and only one episode of menorrhagia was reported [13]. Similar to our study both studies observed bleeding episodes in patients receiving PAC, in particular in relation to aspirin. In contrast to our study, none of these studies included a comparison group, and thus it is not possible to assess if PAC was successful in preventing TE, although the number of observed TE appears to be low compared to the reported incidences in other studies.

Our study is limited by the small study sample size, the retrospective design and the inclusion of only clinically reported TE and bleeding episodes which may underestimate the frequency of both smaller bleeding episodes and subclinical TE. Furthermore, although not significant male sex, a risk factor for TE, tended to be more frequent in the group receiving PAC. This may lead to an underestimation of the preventive effect of PAC when comparing to the group not receiving PAC. In addition, the allocation to PAC were based on different practices in two different nephrology departments implying that other differences in practices may affect the outcome. Furthermore, no algorithm for the selection between Warfarin, LMWH or aspirin was applied. However, except for plasma albumin levels the groups were similar and recruited within the same region of Denmark. Inclusion was based strictly on biochemical criteria and biopsy proven glomerular disease. Given the limited available evidence we believe that the findings provide relevant information to guide PAC in NS.

International guidelines recommend full-dose anticoagulation with LMWH or Warfarin should be considered if serum albumin is below 20–25 g/L in combination with one or more risk factors [17]. Given our findings it is likely that this may prevent TE; however, at the possible costs of increased risk of major and potential fatal bleeding episodes. Thus, careful attention to other factors implying bleeding risks may be recommend and the combination with platelet inhibitors should be avoided.

## Conclusions

This retrospective analysis showed that PAC treatment was associated with a decreased risk of clinically significant TE in patients with NS, but may also be associated with more bleeding episodes, although this difference was not statistically significant between groups. Major bleeding episodes were only observed in patients on concomitant anti-platelet therapy.

## Abbreviations

DVT: Deep vein thrombosis
IQR: Interquartile range
LMWH: Low-molecular-weight heparin
NS: Nephrotic syndrome
PAC: Prophylactic anticoagulation
PE: Pulmonary embolism
RVT: Renal vein thrombosis
TE: Thromboembolic events

## References

[1] Christiansen CF, Schmidt M, Lamberg AL, Horvath-Puho E, Baron JA, Jespersen B, Sorensen HT (2014). Kidney disease and risk of venous thromboembolism: a nationwide population-based case-control study. J Thromb Haemost.

[2] Kerlin BA, Ayoob R, Smoyer WE (2012). Epidemiology and pathophysiology of nephrotic syndrome-associated thromboembolic disease. Clin J Am Soc Nephrol.

[3] Pincus KJ, Hynicka LM (2013). Prophylaxis of thromboembolic events in patients with nephrotic syndrome. Ann Pharmacother.

[4] Llach F (1985). Hypercoagulability, renal vein thrombosis, and other thrombotic complications of nephrotic syndrome. Kidney Int.

[5] Rankin AJ, McQuarrie EP, Fox JG, Geddes CC, MacKinnon B, Scottish Renal Biopsy R (2017). Venous thromboembolism in primary nephrotic syndrome - is the risk high enough to justify prophylactic anticoagulation?. Nephron.

[6] Schlegel N (1997). Thromboembolic risks and complications in nephrotic children. Semin Thromb Hemost.

[7] Mirrakhimov AE, Ali AM, Barbaryan A, Prueksaritanond S, Hussain N (2014). Primary nephrotic syndrome in adults as a risk factor for pulmonary embolism: an up-to-date review of the literature. Int J Nephrol.

[8] Barbour SJ, Greenwald A, Djurdjev O, Levin A, Hladunewich MA, Nachman PH, Hogan SL, Cattran DC, Reich HN (2012). Disease-specific risk of venous thromboembolic events is increased in idiopathic glomerulonephritis. Kidney Int.

[9] Ismail G, Mircescu G, Ditoiu AV, Tacu BD, Jurubita R, Harza M (2014). Risk factors for predicting venous thromboembolism in patients with nephrotic syndrome: focus on haemostasis-related parameters. Int Urol Nephrol.

[10] Gyamlani G, Molnar MZ, Lu JL, Sumida K, Kalantar-Zadeh K, Kovesdy CP (2017). Association of serum albumin level and venous thromboembolic events in a large cohort of patients with nephrotic syndrome. Nephrol Dial Transplant.

[11] Mahmoodi BK, ten Kate MK, Waanders F, Veeger NJ, Brouwer JL, Vogt L, Navis G, van der Meer J (2008). High absolute risks and predictors of venous and arterial thromboembolic events in patients with nephrotic syndrome: results from a large retrospective cohort study. Circulation.

[12] Medjeral-Thomas N, Ziaj S, Condon M, Galliford J, Levy J, Cairns T, Griffith M (2014). Retrospective analysis of a novel regimen for the prevention of venous thromboembolism in nephrotic syndrome. Clin J Am Soc Nephrol.

[13] Rostoker G, Durand-Zaleski I, Petit-Phar M, Ben Maadi A, Jazaerli N, Radier C, Rahmouni A, Mathieu D, Vasile N, Rosso J (1995). Prevention of thrombotic complications of the nephrotic syndrome by the low-molecular-weight heparin enoxaparin. Nephron.

[14] Sarasin FP, Schifferli JA (1994). Prophylactic oral anticoagulation in nephrotic patients with idiopathic membranous nephropathy. Kidney Int.

[15] Stoycheff N, Stevens LA, Schmid CH, Tighiouart H, Lewis J, Atkins RC, Levey AS (2009). Nephrotic syndrome in diabetic kidney disease: an evaluation and update of the definition. Am J Kidney Dis.

[16] Lionaki S, Derebail VK, Hogan SL, Barbour S, Lee T, Hladunewich M, Greenwald A, Hu Y, Jennette CE, Jennette JC (2012). Venous thromboembolism in patients with membranous nephropathy. Clin J Am Soc Nephrol.

[17] Beck L, Bomback AS, Choi MJ, Holzman LB, Langford C, Mariani LH, Somers MJ, Trachtman H, Waldman M (2013). KDOQI US commentary on the 2012 KDIGO clinical practice guideline for glomerulonephritis. Am J Kidney Dis.

